# The impact of vegetative and solid roadway barriers on particulate matter concentration in urban settings

**DOI:** 10.1371/journal.pone.0296885

**Published:** 2024-01-31

**Authors:** Roby Greenwald, Jeremy A. Sarnat, Christina H. Fuller

**Affiliations:** 1 Population Health Sciences Department, School of Public Health, Georgia State University, Atlanta, Georgia, United States of America; 2 Gangarosa Department of Environmental Health, Rollins School of Public Health, Emory University, Atlanta, Georgia, United States of America; 3 University of Georgia College of Engineering, Athens, GA, United States of America; Al Mansour University College-Baghdad-Iraq, IRAQ

## Abstract

A potentially important approach for reducing exposure to traffic-related air pollution (TRAP) is the use of roadside barriers to reduce dispersion from highway sources to adjacent populated areas. The Trees Reducing Environmental Exposures (TREE) study investigated the effect of vegetative and solid barriers along major controlled-access highways in Atlanta, Georgia, USA by simultaneously sampling TRAP concentration at roadside locations in front of barriers and at comparison locations down-range. We measured black carbon (BC) mass concentration, particle number concentration (PNC), and the size distribution of ultrafine aerosols. Our sample sites encompassed the range of roadway barrier options in the Atlanta area: simple chain-link fences, solid barriers, and vegetative barriers. We used Generalized Linear Mixed Models (GLMMs) to estimate the effect of barrier type on the ratio of particle concentrations at the comparison site relative to the roadside site while controlling for covariates including wind direction, temperature, relative humidity, traffic volume, and distance to the roadway. Vegetative barriers exhibited the greatest TRAP reduction in terms of BC mass concentration (37% lower behind a vegetative barrier) as well as PNC (6.7% lower), and sensitivity analysis was consistent with this effect being more pronounced when the barrier was downwind of the highway. The ultrafine size distribution was comprised of modestly smaller particles on the highway side of the barrier. Non-highway particle sources were present at all sample sites, most commonly motor vehicle emissions from nearby arterials or secondary streets, which may have obscured the effect of roadside barriers.

## Introduction

The public health consequences of ambient particulate matter (PM) have been well-documented by more than three decades of epidemiologic, toxicologic and clinical studies. The global burden of disease attributable to ambient PM is at a historical high and is estimated to be greater than 5 million deaths per year, including an estimated 108,000 deaths in the United States [1]. In addition to increases in mortality, air pollution exposure is associated with adverse cardiovascular, respiratory and neurological outcomes and diseases. Cardiovascular effects identified through epidemiological studies include myocardial infarction, atherosclerosis, and heart rate variability [2]. Respiratory effects associated with exposure include pulmonary infections, cough, wheeze and chronic obstructive pulmonary disease (COPD). There is sufficient evidence of a causal association with asthma incidence and severity among children [3]. Recent evidence has shown that PM impacts the neurological system triggering the development of Alzheimer’s disease and autism in adults and children, respectively [4, 5]. Sensitive subpopulations, due to existing morbidities or marginalization by race, class or income, may be at increased levels of susceptibility compared to the general population [6–8].

Although air quality has improved in recent decades in the United States, there is substantial variation in exposure based on proximity to sources. Hot spots of exposure are present close to highways and busy roadways [9]. Urban air pollution is frequently dominated by traffic-related air pollution (TRAP) produced by the burning of diesel and gasoline fuel in trucks, buses and cars. Areas near highways and major roads have elevated levels of TRAP compared to areas that are further away from these mobile sources [10–12]. The distribution and down-range gradient of PM from traffic corridors in the near-road environment is not identical for all PM size fractions [11, 13]. Concentrations of ultrafine particles (UFP: < 0.1 μm in aerodynamic diameter) exhibit the steepest gradients. UFP are often estimated in exposure studies by particle number concentration (PNC), which is an effective proxy. We will therefore utilize the PNC measurement for the remainder of this paper. In addition, elevated PNC levels can extend further distances from roadways depending on local meteorological conditions, such as the height of the planetary boundary layer and temperature. In some circumstances, elevated PM concentrations have been found up to 2600 m downwind of highways [14, 15]. Solid carbonaceous particles (black carbon, BC) also exhibit steep gradients, while fine mode PM (PM with aerodynamic diameter less than 2.5 μm, PM_2.5_) shows a very shallow gradient [10].

Scientific evidence has shown that PNC and BC are also associated with adverse health effects, like emergency department visits and elevated cardiovascular risk, independent of those of PM_2.5_ and PM_10_. Neither PNC nor BC are directly regulated by the United States Environmental Protection Agency, and therefore, risk due to exposure may not be controlled by existing standards for PM [16]. Approximately 45 million people in the United States live within 300 m of a major highway, which constitutes a sizable amount of the population being at risk [17].

It is imperative to investigate specific solutions that address elevated concentrations of pollutants in high exposure transportation corridors. Green infrastructure is one mitigation measure for pollution reduction. A recent report from the World Health Organization (WHO) advised further evaluation and implementation of green infrastructure as one avenue to improve health of urban residents [18]. Green infrastructure has a broad definition that may include various forms and functions. The type of green infrastructure discussed here includes existing or planted stands of vegetation alongside roadways [19–23]. Roadside vegetation has been shown to reduce ambient PM concentrations by increasing dispersion, interfering in pollutant transport and direct interception of particles [19, 20, 24, 25]. Other studies have measured increases in concentrations on road and in adjoining areas [20]. The efficacy of vegetation in reducing PM also varies across the year [16]. These apparent discrepancies stem from the fact that vegetation are living organisms that are in constant interplay with the environment around them. Vegetation absorbs carbon dioxide and releases oxygen along with other chemicals such as biogenic volatile organic carbons (BVOCs). A portion of these chemicals are the source of naturally created PM.

Very little is known about the impact of green infrastructure on the chemical composition of particulate matter or the physiological effects of exposure. It is possible that PM that has passed through vegetation may have more benign or more harmful impacts on the human body. Combining the knowledge we currently have there are some basic characteristics that separate a ‘good’ barrier from a ‘poor’ one; however, more research is necessary to optimize a vegetative barrier for a specific location or set of conditions [21, 26].

This study was designed to provide data that fills gaps regarding the impact of vegetative barriers on near-roadway PM concentrations as well as the effect of PM on human cells. We have explored multiple sampling sites in a single metropolitan area (Atlanta, Georgia, USA) to compare and contrast roadside barrier types and meteorological conditions. A novel aspect of this study was that we utilized generalized linear mixed models (GLMMs) to estimate the effect on PM concentration of different types of roadside barriers while controlling for covariates including meteorological and environmental parameters. GLMMs are a widely-used multivariate modeling approach including both fixed and random effects to produce effect estimates of grouped data [27].

The Atlanta metropolitan area has a total population of 4.7 million people. It contains four large controlled-access interstate highways (I-20, I-75, I-85 and I-285) and a major state-level controlled-access highway (GA-400). In 2019, the regional roadway network carried 151 million vehicle miles traveled (VMT), of which 50 million VMT where on controlled-access highways. The most-traveled highway segment is the co-listed I-75/85 through downtown Atlanta with 352,000 daily VMT. All traffic data is from the Georgia Department of Transportation [28]. We describe here the findings of continuous PM concentrations from the multiple sites, while toxicological impacts of exposure are given in a separate paper.

## Methods

### Site selection

**Fig 1:**
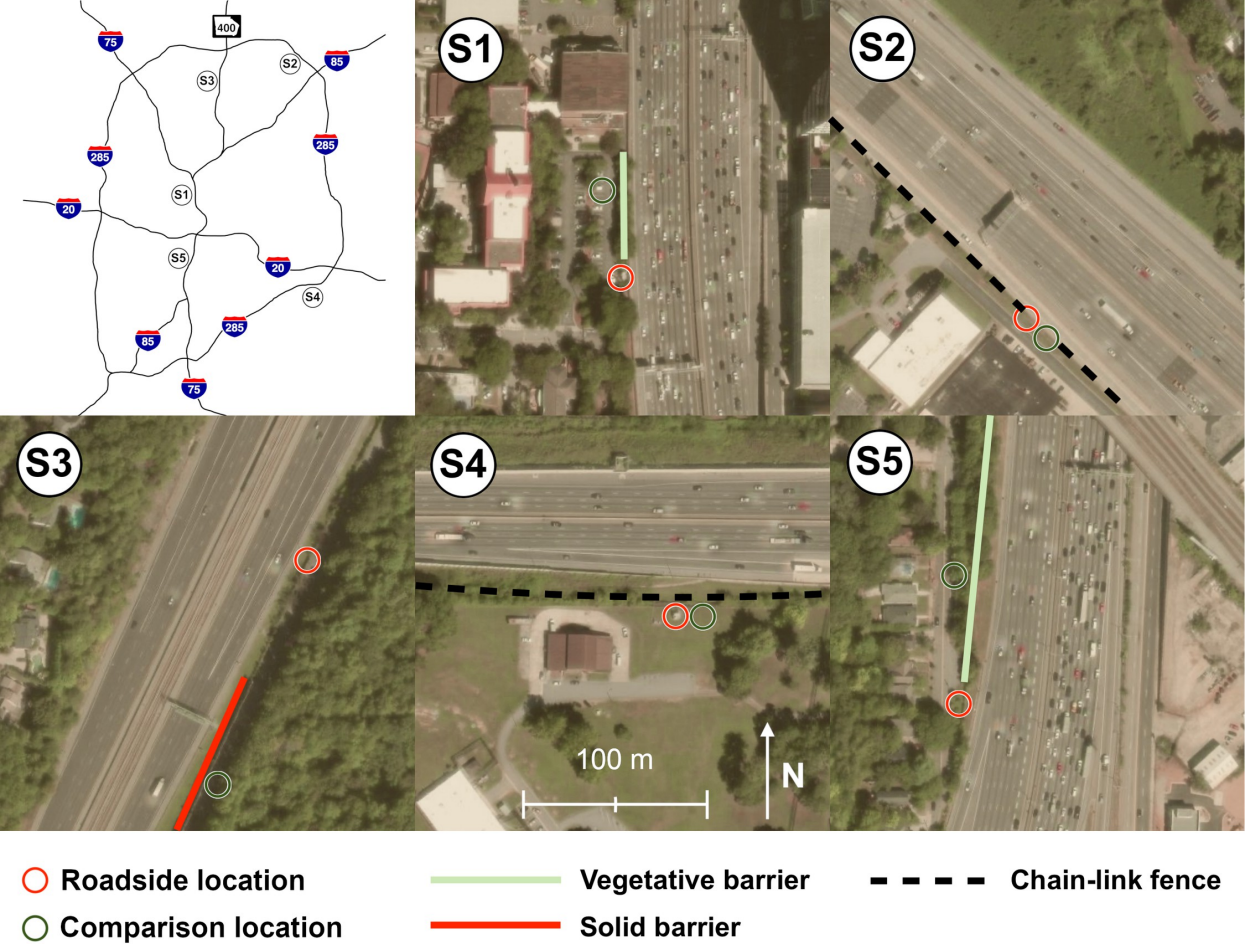
Aerial view of sample sites. All images are to the same scale and oriented with north being up.

We selected five sample sites in the Atlanta metropolitan area. Sites are shown in Fig 1 and a summary of site characteristics is given in Table 1. All sites were adjacent to a high-volume controlled-access highway. Given that all sampling was conducted on private property abutting the public right of way, no special permits were required from the Georgia Department of Transportation. All such highways in Georgia are separated from adjacent areas by a physical barrier to prevent pedestrian or unauthorized vehicle entry. These barriers are typically chain-link fences but in urban areas may instead be a solid sheet metal barrier for noise abatement. Selected sample sites include two with vegetative barriers, one with a solid barrier, and two with only a chain-link fence. Vegetative barriers consisted of multiple tree species, typically dominated by magnolias, water oaks, loblolly pines, and crepe myrtles, and vegetative barrier sites also include a chain-link fence. Sampling occurred during the summer season with all vegetation in full foliage. Given that chain-link fences have a negligible effect on particle concentrations, we included chain-link fence sites as a control group for comparison purposes. However, it should be noted that under summer conditions in Georgia, fast-growing vine and grass species can grow along chain-link fences in between road maintenance intervals and constitute a minimal amount of vegetative coverage. We used a dual location approach to make simultaneous comparisons of pollutant concentrations at the roadside and behind the barrier at each of the five sample sites. Each sample site therefore had a “roadside” location, which was as close to the roadside as permitted by Georgia Department of Transportation rules (7–19 m from the pavement edge), and a “comparison” location, which was behind the roadside barrier. We could not quantitatively measure lateral leaf area density; however, we used surface photos and satellite imagery to qualitatively assess vegetation density in five categories ranging from no vegetation other than grass to full vegetation to a 10 m height with no visible gaps.

**Table 1:** Sample site characteristics.

Site code	adjacent highway	barrier type	pavement edge to roadside location [m]	pavement edge to comparison location [m]	direction of highway from sample site	average vehicles per hour^a^	qualitative vegetation density
							category^b^
S1	I-75/85	vegetative	7	19	E	19,700	5 (2019)
						(4,520)	4 (2020)
S2	I-285	chain-link fence	8	8	NE	13,500	1
						(830)	
S3	GA-400	solid	18	22	WNW	7,990	2 (2019)
						(1,490)	1 (2020)
S4	I-285	chain-link fence	19	29	N	7,300	1
						(780)	
S5	I-75/85	vegetative	7	18	E	15,300	3
						(2,040)	

^a^ Values are mean(SD) during sampling periods, data from Georgia Department of Transportation

^b^ 1 –no vegetation, 2 –some vegetation with gaps comprising more than 50% of horizontal distance, 3 –vegetation gaps less than 50% of horizontal distance, 4 –vegetation covering the full horizontal distance but small visible gaps in the vertical direction, 5 –minimal visible gaps in vegetation

### Instrumentation

We measured the physical, chemical, and toxicological properties of ambient PM using both integrated and continuous approaches, and in this paper, we present the results from continuous instrumentation. We used a TSI, Inc. Model 3330 Optical Particle Sizer to measure fine-mode PM number concentration in 16 size bins with 1-minute time-resolution. Only one of these instruments was used for this protocol, and we operated this instrument exclusively at the roadside. As a proxy for mass concentration, we calculated the volume concentration of each size bin by assuming all particles were spheres with a diameter equal to the logarithmic mean of the upper and lower limits of the bin. We then calculated the PM_1_ and PM_2.5_ volume concentration by summing the volume concentration of the bins smaller than 1.0 μm and 2.5 μm respectively.

We used TSI, Inc. Model 3910 NanoScans to measure the particle number concentration (PNC) and size distribution with 1-minute time-resolution, including the geometric mean diameter (GMD) and geometric standard deviation (σ_G_), in the ultrafine size mode. In year 1, we used a pair of NanoScans with one at the roadside and the other the comparison site. However, one of the pair was not available during year 2, and the remaining unit was used exclusively at the roadside. As an alternative measure of UFP, we used a pair of TSI, Inc. Model 3007 Condensation Particle Counters (CPC) to measure total PNC, both at roadside and the comparison site.

We measured the mass concentration of particulate black carbon (BC) using Aethlabs Model AE51 microaethalometers. In year 1, we used four units, two at roadside and two at the comparison site while in year 2, we used one unit at roadside and one unit at the comparison site. These devices were configured to log both reference and sensor signal data with a time-resolution of 1-second, and we calculated BC mass concentration with 1-, 5-, and 10-minute resolution with adjustment for aethalometer filter loading as described by Good *et al*. [29].

We measured meteorological parameters including temperature, relative humidity, wind speed, wind gusts, and wind direction with 1-minute time resolution using several weather stations including an Onset Computer HOBO U30, and both a Vantage Pro and Vantage Vue model produced by Davis Instruments. A weather station was placed at the roadside sampling location and another at the comparison sampling location. For each minute of data collection, we calculated wind direction relative to the highway using the roadside monitor such that a wind direction of 0° corresponded to the sample site being directly downwind of the highway, a wind direction of 180° corresponded to the site being directly upwind of the highway, and a wind direction of 90° indicated that the wind was parallel to the highway. We then created four categorical variables for wind direction: a calm wind category of wind speed <0.1 m/s, wind from the highway for relative wind direction <45°, wind parallel to the highway for relative wind direction 45°-135°, and wind toward the highway for relative wind direction >135°.

At the beginning and end of each sampling session, all continuous instruments were co-located for 10–30 minutes at either the roadside or comparison location. For each of the instrument types, we chose one unit as the reference, and in all cases, this reference unit was present at all sampling sessions. We calculated the percent difference between each unit and the reference unit for each type of instrument. We performed linear regression between all instruments with the corresponding reference unit and used these regression coefficients to adjust the data relative to the reference unit.

### Data analysis

We aggregated all continuous data to 5-minute time intervals. We calculated summary statistics for all pollutant data and for strata for each sample site and wind direction category. To estimate the effects of roadside barriers as well as meteorological parameters, we used GLMMs, specifically the multivariate penalized quasi-likelihood (glmmPQL) function of the nlme package for R v4.1.2 (R Foundation for Statistical Computing). We included a random effect for date and a temporal autocorrelation structure based on the chronological order of the observations, specified as corAR1(form = ~1). The dependent variable in each model was the ratio of the pollutant measured at the comparison site to that measured at the roadside. The four parameters evaluated using this approach are PNC (using NanoScan in year 1 and CPC data in year 2), GMD and σ_G_ (using NanoScan data, which was only available at both sites in year 1), and BC (available in both years). We began with the full model for each pollutant parameter including an intercept, the baseline quantity (*i*.*e*. the parameter measured at the roadside), ambient temperature, relative humidity, wind speed, and wind direction category (all meteorological variables as measured at the roadside), the interaction of wind speed and wind direction, traffic counts, distance to the roadside sample location, distance to the comparison sample location, and a categorical variable describing the barrier type. We performed a backwards stepwise model selection process by eliminating variables that had minimal effect on the dependent variable, and this process removed wind speed as well as the interaction of wind speed and wind direction. We conducted sensitivity analysis by evaluating the final models separately for each wind direction category and barrier type.

## Results

### Nature and variation of vegetative barriers

Given that the height, depth and species of vegetation has been shown to be important to near-roadway PM associations [20], we selected five distinct locations with site characteristics that displayed diversity in the form and nature of the vegetation. Sampling occurred during the summer season with all vegetation in full foliage. The vegetative barrier at S1 extended for 250 m parallel to the highway with 15 m gap for state-owned roadside monitors. We sampled 15 m north of this gap. The vegetative barrier at S5 is 200 m parallel to the highway, and we sampled 15 m north of the southern end of it. The depth of the vegetation perpendicular to the roadway was the same at both sites (essentially the width of one mature pine or magnolia tree). The vegetative barrier site at S1 was approximately 10 m in height, while site S5 was about 5 m in height. Some trimming had occurred at S1 between sampling seasons such that vegetation density was noticeably less dense, nonetheless, both sites had either tree branches reaching all the way to ground level or understory vegetation extending up to the canopy. Site 3 (the solid barrier site) had numerous small pine trees in front of the barrier in 2019, but these were removed before sampling in 2020.

### Side-by-side comparison of continuous instrumentation

When using co-located data aggregated at 5-minute time intervals, we found the percent difference between each unit of microaethalometer and the reference unit to be 16% (31%) and -0.91% (20%) for the two units used only in year 1 and -5.1% (25%) for the unit used in years 1 and 2. We observed a mean (SD) difference in PNC between NanoScan units of 3.3% (8.0%), 1.8% (5.0%) in GMD, and 0.38% (3.1%) in σ_G_. We compared PNC data as measured by the CPC instruments to the reference NanoScan and found a 5.1% (37%) difference for one unit and a 25% (45%) difference for the other. Although one of the CPC units showed less agreement with the reference NanoScan, it is important to note that all instruments were unbiased after adjustment to the reference unit using linear regression coefficients.

### Stratification by barrier type and by wind direction

**Fig 2:**
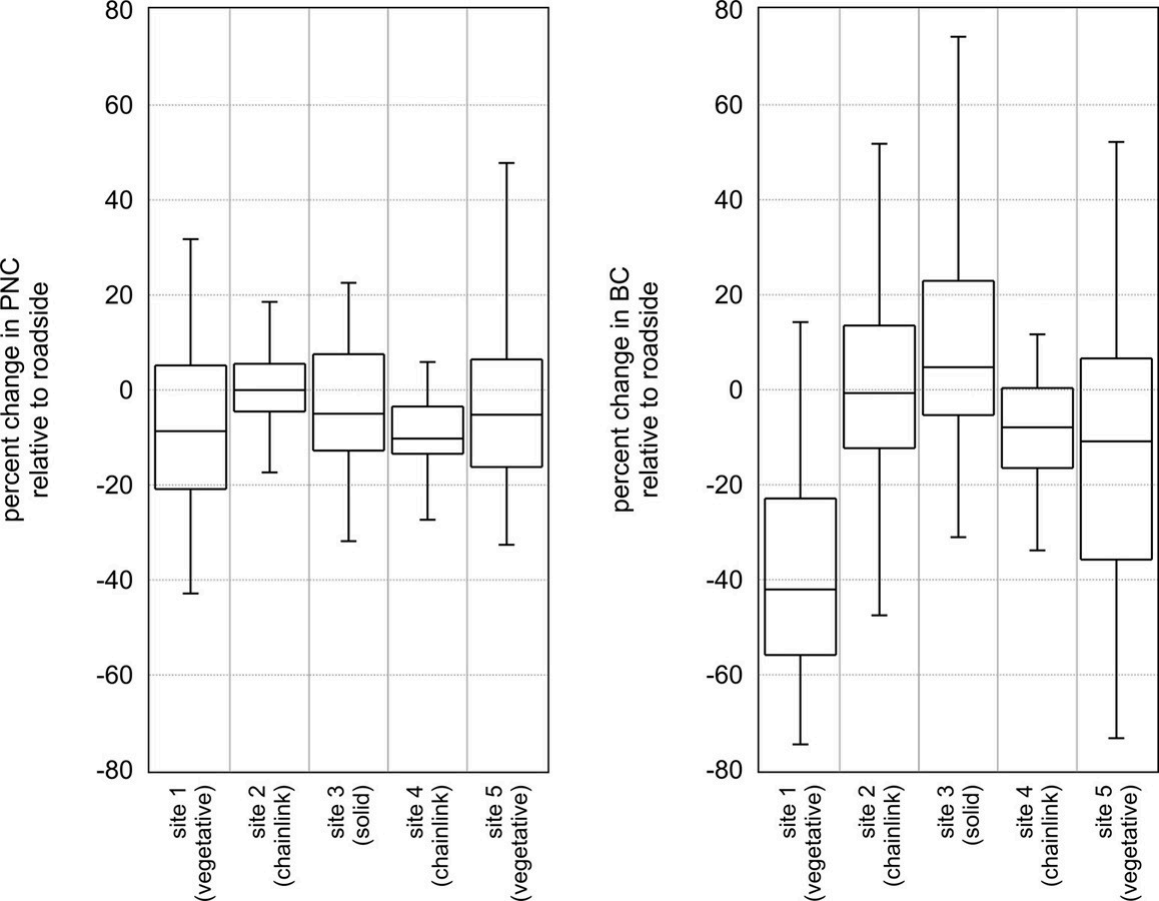
Summary statistics of the percent difference in PNC (left) and BC (right) data relating comparison locations to roadside locations stratified by sample site. Boxes are the first quartile, median and third quartile, and whiskers are the 5^th^ and 95^th^ percentiles.

We examined summary statistics for the change in pollutant levels for strata of sampling site and wind direction category. The summary statistics of the percent difference in PNC and BC data relating the comparison location to the roadside location stratified by sample site are shown in Fig 2. For PNC, the median percent difference including data from all sites and wind conditions was -3.0% with an interquartile range (IQR) of 18%. For BC, the median percent difference was -7.5% (IQR = 44%). In both cases, this indicates that the median concentration at the comparison location was slightly lower than at the roadside. When stratifying by site, we observed the median percent difference to be negative at all sites. The median percent difference was near zero for S2, but was 5–10% lower at all other sites, including sites with vegetative and solid fence barriers. This was an expected finding given that a key distinction for S2 is that there is only a chain-link fence at this site and both the roadside and comparison sampling locations were the same distance from the roadway (they were separated from each other by 20m parallel to the roadway). S4 also only has a chain-link fence but curiously shows a median reduction in PNC of 10%. Two features of this site are that the comparison location was at a slightly lower elevation (~1m) compared to the roadside location and that the chain-link fence in front of the comparison location was thinly covered by a fast-growing vine species that was present on some sample days but not others. Both of these features would be expected to have a modest negative effect on PNC at the comparison location. For BC, we observed a wider distribution of difference in concentration. The reduction in BC was higher at the sites with a vegetative barrier than at sites with a chain-link fence or solid barrier. The median change was 42% lower at S1, near zero for S2 (similar to PNC), 5% higher at S3, 8% lower at S4, and 11% lower at S5. The reduction was much higher at S1 than the other sites, and an important distinction for S1 is that it was adjacent to the roadway segment with the highest traffic volume of any sample site. The median difference was positive for S3, and a key distinction of this site is that it has the lowest traffic volume of all the sites, and furthermore, as it is adjacent to GA-400, which is predominantly a commuter highway with restrictions on commercial truck traffic, we observed lower BC concentrations at this site (1.4 μg·m^-3^ compared to 3.0–4.6 μg·m^-3^ for other sites).

**Fig 3:**
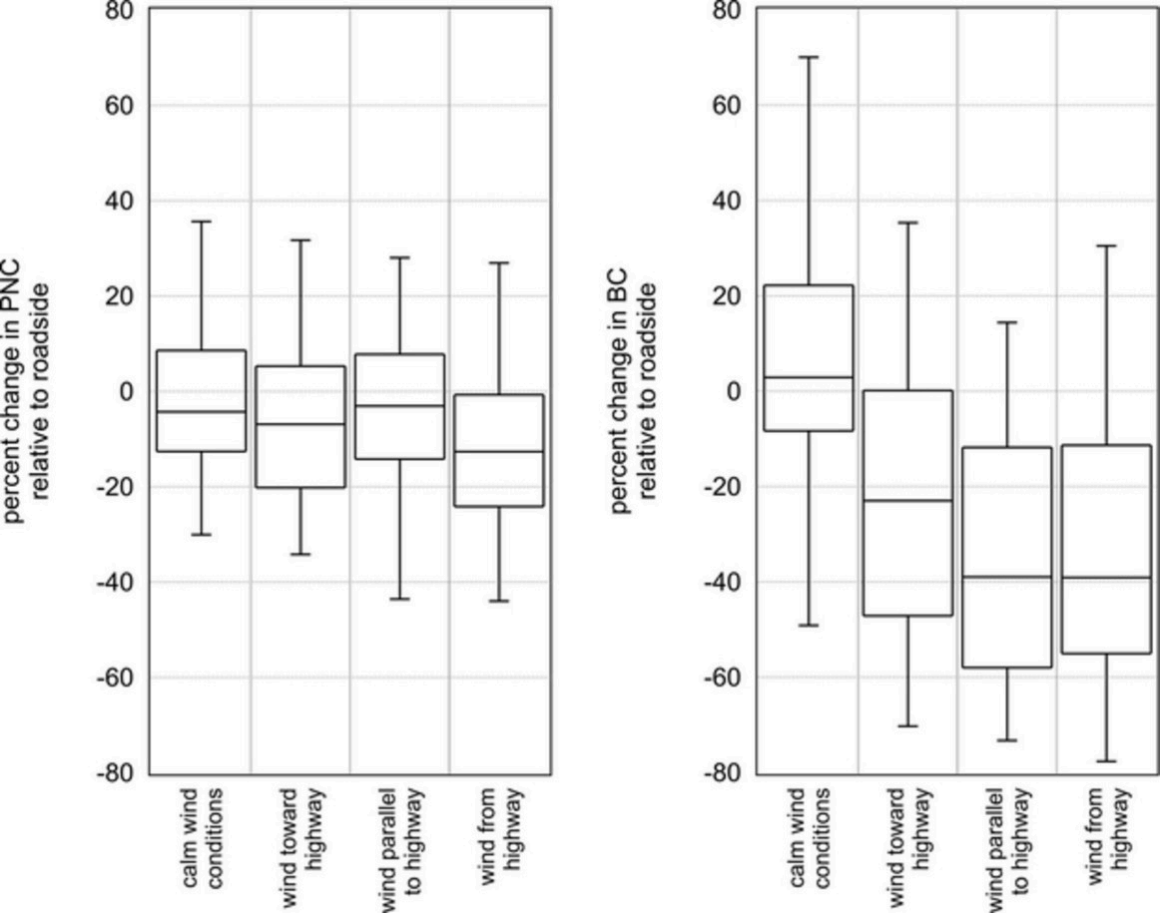
Summary statistics of the percent difference in PNC (left) and BC (right) data showing the percent difference between the comparison locations and the roadside locations stratified by wind direction category. Sites with only a chain-link fence are not included (S2 and S4). Boxes are the first quartile, median and third quartile, and whiskers are the 5^th^ and 95^th^ percentiles.

The summary statistics of this same data stratified by wind direction category are shown in Fig 3. Given that chain-link fences have a negligible effect on particle dispersion, Fig 3 does not include data from chain-link fence sites (S2 and S4). For PNC, the percent difference is negative for all categories, and the magnitude of the change is inversely proportional to the relative wind direction. In other words, when the comparison location is upwind of the highway, PNC is modestly lower at the comparison location, but when it is downwind of the highway, the reduction in concentration is more pronounced. For BC, the range of observed differences in concentration is substantially wider than for PNC. Under calm wind conditions, the median percent difference in BC concentration is slightly positive (2.9%). When the sample site is upwind of the highway, median BC concentration is 23% lower at the comparison site, whereas when the wind direction is either parallel to the highway or the sample site is downwind, the median percent difference is -39%. It should be noted that there is substantial overlap in the stratification categories in that the overwhelming majority (81%) of observations at site 3 (solid barrier) occurred under calm wind.

### Multivariable modeling results

Table 2 shows the multivariable GLMM results. The dependent variable is the ratio of pollutant parameters at the comparison location to those at the roadside location. The backwards stepwise model selection process resulted in the final model including baseline quantity (*i*.*e*., pollutant parameter as measured at the roadside), ambient temperature, relative humidity, traffic counts, distance to the roadside sample location, distance to the comparison sample location, wind direction category, and categorical variables describing the barrier type. The barrier type categorical variables use the chain-link fence as the reference category, and the wind direction categorical variables use the calm wind conditions as the reference category. An alternative set of models used a qualitative categorical variable describing vegetation density in the place of the barrier type categorical variable. It was not possible for both variables to be included simultaneously due to their high correlation. The reference category for this variable was category 1, no vegetation other than grass.

**Table 2:** Results of general linear mixed models (GLMM). The dependent variable in each model is the ratio of the pollutant parameter measured at the comparison location to that measured at the roadside location. Categorical variables for wind direction use calm wind conditions as the reference category and barrier type categorical variables use the chain-link fence barrier as the reference. The first row in each box is the parameter estimate while the second and third rows are the lower and upper 95% confidence intervals (respectively).

pollutant parameter	vegetative barrier	solid barrier	baseline quantity	temperature [°C]	relative humidity [%]	traffic volume [vehicles per hour]	pavement edge to roadside site [m]	pavement edge to comparison site [m]	wind direction from highway	wind direction parallel to highway	wind direction toward highway
PNC	0.080	-0.072	-7.2E-6	-0.015	0.0011	-1.8E-4	0.030	3.8E-4	-0.19	-0.14	-0.10
	-0.052	-0.21	-1.1E-5	-0.028	-0.0029	-4.2E-4	0.013	-0.0032	-0.30	-0.24	-0.22
	0.21	0.071	-3.2E-6	-0.0023	0.0050)	6.8E-5	0.046	0.0040	-0.081	-0.039	0.013
GMD	0.22	0.11	-0.0050	0.021	0.0058	2.4E-4	-0.029	-1.2E-4	-0.0024	-0.013	0.069
	-0.31	0.034	-0.0062	0.015	0.0038	1.4E-4	-0.073	-0.0012	-0.042	-0.049	0.011
	0.75	0.18	-0.0039	0.027	0.0078	3.4E-4	0.014	9.7E-4)	0.038	0.022	0.13
σ_G_	0.30	0.11	-0.0088	0.022	0.0056	2.8E-4	-0.039	-2.1E-4	-0.0098	-0.032	0.012
	-0.32	0.027	-0.010	0.016	0.0032	1.6E-4	-0.090	-0.0015	-0.056	-0.074	-0.056
	0.91	0.20	-0.0071	0.029	0.0079	3.9E-4	0.012	0.0011	0.037	0.0092	0.079
BC	-0.23	0.21	-0.012	0.025	0.0044	-1.2E-4	0.016	9.3E-4	-0.15	-0.068	-0.11
	-0.37	0.016	-0.021	0.013	0.0012	-3.2E-4	0.010	-0.0044	-0.26	-0.17	-0.24
	-0.094	0.41	-0.0022	0.036	0.0076	7.3E-5	0.042	0.0063	-0.038	0.033	0.089

The parameter estimates for PNC were not statistically significant for either barrier type when controlling for other co-variates. The estimated change in the ratio of PNC at the comparison site to the roadside site was slightly positive (β = 0.080) for the vegetative barrier using a chain-link fence as the reference, though the 95% confidence intervals include zero (p = 0.234). For solid barriers, the estimated change in the PNC ratio was slightly negative (β = -0.072) though also not significant (p = 0.325). The PNC model estimates for wind direction are consistent with numerous previous findings of PNC being reduced with distance downwind from highways [20, 30]. In other words, using calm wind conditions as the reference, the parameter estimates for wind direction categorical variables indicate the greatest statistically significant reduction in PNC ratio when the sample site is downwind of the highway (β = -0.19, p = 0.00075), a smaller reduction when the wind is parallel to the highway (β = -0.13, p = 0.0063), and a non-significant difference when the sample sites are upwind of the highway (β = -0.10, p = 0.081). In addition, the PNC model parameter estimate for the distance from the pavement edge to the roadside sample location is positive and significant (β = 0.030, p = 0.00066), indicating that as distance from the roadway increases, the ratio of comparison location to roadside location PNC also increases. This is consistent with previous findings that PNC decrease down-range of a highway is non-linear [30].

For the ultrafine mode size distribution parameters of GMD and σ_G_, the parameter estimates for both barrier types were positive, though only those for the solid barrier were statistically significant. Given that smaller sized particles have higher Brownian diffusion rates and thus higher dry deposition velocities, it was expected that the reduction in concentration for ultrafine particles would be inversely correlated with particle size. The overall reduction in PNC as well as a shift in the distribution toward larger sizes is consistent with this expectation that changes in PNC are the result of disproportionate deposition of smaller sized particles on roadside surfaces including vegetation.

For the change in the ratio of BC concentration, the parameter estimates were statistically significant for both barrier types, though in opposite directions. The estimated change was negative for the vegetative barrier (β = -0.23, p = 0.0010), indicating the reduction in BC concentration was higher for vegetative barriers than for chain-link fences when controlling for other co-variates. On the other hand, the estimated change for solid barriers was positive (β = 0.21, p = 0.034). For wind direction categories, the model estimates for BC are similar as for PNC with the exception that wind parallel to the highway exhibited somewhat less of a reduction in BC concentration than wind perpendicular to the highway. Similar to that found for PNC, the BC model parameter estimate for distance to the roadway was positive and statistically-significant.

We conducted sensitivity analysis for the vegetative barrier variable using wind direction categories other than calm wind conditions. For the wind from the highway category, the parameter estimate for the vegetative barrier was -0.091 (p = 0.62) for PNC and -0.94 (p = 0.0049) for BC. For the wind parallel to the highway category, the parameter estimate for the vegetative barrier was 0.022 (p = 0.63) for PNC and 0.049 (p = 0.58) for BC. For the wind toward the highway category, the parameter estimate for the vegetative barrier was 0.51 (p = 0.0091) for PNC and -0.12 (p = 0.55) for BC. Given that calm wind conditions were predominant at the solid barrier site while there were few calm wind observations at the chain-link fence sites, sensitivity analysis for wind direction category did not yield meaningful parameter estimates for the solid barrier type due to a lack of statistical power.

The results of alternative models using a qualitative vegetation density categorical variable were similar to model results using the barrier type categorical variable. For BC, the largest reduction in concentration was for denser vegetation (β = -0.44, p = 0.042 comparing category 5 to category 1 and β = -0.47, p = 0.030 comparing category 4 to 1). For PNC, the parameter estimates for vegetation density were smaller and not statistically-significant. Given the high correlation between vegetation density and barrier type, it was not possible to evaluate both variables in the same model. However, we have chosen to focus on barrier type models because these models more plausibly include a distinction between solid barriers and chain-link fences whereas vegetation density models do not.

## Discussion

In this study, both vegetative and solid barriers were associated with changes in particle concentrations when compared to locations with only a chain-link fence. We found associations between wind direction and particle concentrations, and we found that the effect of the barriers was highest when they were downwind of the highway. The observed particle reductions were more pronounced for BC than for PNC. Some of this difference is likely due to differences in sources between types of particles. BC is entirely combustion-sourced, and primarily influenced by roadway emissions during this study. PNC on the other hand has both anthropogenic and natural sources and is influenced by roadway emissions and other anthropogenic sources as well as natural biogenic emissions. For PNC, the mean concentration at the comparison site was 8.5% lower than the roadside when downwind of the highway and was 2.6% lower when upwind. For BC, the mean concentration at the comparison site was 23% lower when downwind and 10% lower when upwind. We conducted monitoring campaigns in the summer with full foliage and warm temperatures, and our results are only representative of this time range. This time period was selected to evaluate effects of vegetation with the greatest density of foliage. Other seasons may differ due to changes in the amount of foliage as well as ambient temperatures.

A majority of studies through 2017 reported reductions in pollutant concentrations between 15% and 60% as noted in a review Abhijith *et al*. [20]. For those studies quantifying BC and PNC, several found reductions on the order of 10–65%. Black carbon concentrations were reduced up to 12.4% under downwind wind conditions in a study of a mixed species tree stand [31]. In that study there was a reduction in BC of 7.8% under parallel wind conditions and 5.9% when winds were low [31]. Abhijith *et al*. [24] measured BC concentrations under six configurations of vegetative barriers. The configuration most similar to ours (instruments placed alongside and behind trees and hedges) reported a 4% reduction in BC and a 2% reduction in PNC, while a reduction of 63% for BC and 30% for PNC was measured comparing in front of and behind a row of trees and hedges when the wind direction was parallel to the roadway. Black carbon concentrations were reduced by 12.4% in a sampling study of a mixed species tree stand when the site was downwind of the road.

A notable strength of this study was our use of GLMMs to estimate the effect of roadside barriers on particulate concentrations while controlling for covariates including wind direction, traffic volume, and distance to the highway. To our knowledge, this was the first field study to use GLMMs for this purpose. Importantly, this approach afforded us the ability to estimate the effect of covariates while controlling for barrier types. The effect estimate for wind direction was lowest for calm wind conditions and was highest when the comparison location was downwind of the highway when controlling for other covariates. This is consistent with both our own results stratified by wind direction category (Fig 3) as well as with previous findings by Hagler *et al*. [32], one of the few roadside barrier studies reporting results stratified by wind direction.

Sensitivity analysis reinforced these findings for vegetative barrier sites. For both PNC and BC, the reduction in concentration was most pronounced when the wind was blowing from the highway. This trend may be due to that when the barrier is upwind of the highway, the contribution of non-highway sources was more pronounced, which intuitively obscures the effect of the barrier. When wind direction was parallel to the highway, both highway and non-highway sources impacted the barrier site, and our results were less consistent under these conditions.

Although sample sites for this study were adjacent to high-volume highways with traffic counts of at least 7000 vehicles per hour, these sites were also impacted by emissions from other sources. Motor vehicle emissions are among the largest contributors to air pollution in the Atlanta-region, but other important sources include emissions from biomass burning, secondary organic aerosols (SOA), and sulfate aerosol from regional coal-fired power plants [33–35]. During the time periods sampled for this study, the most important non-roadway source affecting the difference in particle concentrations between the roadside and comparison sites was SOA. Despite its potential to contribute as a non-highway particle source, SOA does not contain elemental carbon and therefore has little influence on BC observations; however, it could potentially have some impact on PNC. Previous studies have found the size distribution of SOA to be approximately 0.4–1.0 μm [36, 37]. Although this size range is larger than that measured by the TSI Model 3910 NanoScan, the tail of the distribution on the small end may extend into the ultrafine mode and lead to modest increases in PNC.

Another likely non-highway source of particles is motor vehicle emissions from other roadways in addition to the adjacent highway. All sites were near low traffic volume secondary streets, and site 1 was approximately 50 m from a bus stop on a secondary street that was serviced by a diesel-powered bus at intermittent intervals. Site 2 was 480 m from a large arterial (Buford Highway, approximately 2000 vehicles/hour during the sampling time period) and 900 m from both a heavy rail transit line (MARTA, Doraville station) and a freight rail switching yard (Norfolk Southern, Chamblee yard). Although these distances are large compared to the distance to the highway, they are not atypical for urban settings, and it is likely these sources had a non-zero influence on particle concentrations at site 2. In any case, non-highway particle sources have the effect of obscuring the impact of the barriers.

All sampling sites were located as close to the highway as possible; however, it is worth highlighting that their proximity to the traffic sources did vary among the sites. In addition, we attempted to keep the distance between the roadside and comparison sites consistent at all locations, but we were constrained in some cases by property lines such that the two sampling sites were closer to each other at some locations. At site 2 (chain-link fence), the roadside and comparison sites were the same distance from the pavement edge, and at site 3 (solid barrier), the comparison site was only 4 m further from the pavement than the roadside site. Given that particle concentrations exhibit exponential (as opposed to linear) decay with distance to the roadway [30], the ratio of particle concentration at the comparison site to the roadside site would be expected to differ based on both distance to the highway and distance between sites. In order to control for differences in sampler proximity to both the roadside and comparison sites as well as to assess the independent effects of other covariates, we included explicit terms for distance in our analytical approach using GLMMs. Despite these efforts to control for distance, we acknowledge differential proximity to the roadways as a potential, residual source of uncertainty that may explain some of the differences in our reported observations. In addition, it is important to note that the lengths of our barriers (the distance parallel to the highway) were relatively short. Previous studies have noted the impact of eddy effects along the edges of barriers whereby roadway particles are able to wrap around behind the barrier. This may have occurred during our monitoring and yielding higher concentrations behind the barrier at the locations we monitored.

By coincidence, we performed one season of sampling in the summer of 2019 before the COVID-19 pandemic occurred and the second season in summer 2020, during the initial phase of the pandemic that involved extensive stay-at-home policies in the U.S., including Atlanta. Traffic volumes in this region were more than 10% lower in 2020 than in 2019 which resulted in a larger dynamic range for the observed traffic volumes than would have otherwise occurred. Interestingly, the model estimates for the effect of traffic volume were largely null, consistent with the effect of barriers being proportionally similar for high and low volume roadways.

We observed mixed results at site 3 (which included a solid barrier) in both 2019 and 2020. For BC, the direction of the change in concentration relative to the roadside was opposite to that observed at the vegetative barrier sites. There are a number of features of this site that could potentially contribute to these findings; however, we were unable to evaluate which, if any, of these features contribute to our unexpected findings. First, the vast majority (81%) of observations at site 3 occurred under calm wind conditions, for which it was not possible to determine wind direction. Consequently, the solid barrier categorical variable was highly correlated with the calm wind conditions categorical variable, and it was not possible to infer the causal influence of the solid barrier. Second, this site was adjacent to GA-400, which is a controlled-access freeway utilized disproportionately by light passenger vehicles with comparatively fewer diesel commercial trucks, implying lower BC emissions from the highway in this location. This is consistent with our observation that the median roadside BC concentration at this site was less than half that of any other site. This could increase the impact of non-highway BC sources in the vicinity of this site (including the adjacent secondary road), which would have the effect of increasing the ratio of comparison to roadside BC at this site. Third, the sampling location at this site was along a 2-lane secondary road and in between a 6m solid metal wall and a row of 20m pine trees. The location of the comparison site in a virtual trough between a wall and a row of trees could reduce the ability of highway emissions to reach the near ground-level instrumentation, which would again increase the impact of non-highway sources.

This analysis included design limitations that may be addressed in future projects examining roadside vegetative barriers. Although this study included more monitoring sites than the majority of studies of this type, the variation between sites made comparisons challenging. We selected 5 sites in the Atlanta region based on several criteria including the presence of barriers of various types and the ability to safely operate equipment for 4–6 hours on both sides of the roadway barrier. With additional resources, a larger number of sites could be sampled to provide additional statistical power for assessing the impact of barriers and other covariates. Second, the conditions at the sample sites were controlled by various state and local agencies such that trimming or cutting of vegetative barriers occurred between sampling visits. Site 1 had more dense vegetation in year 1 than in year 2, and site 3 (the solid barrier site) had vegetation present in year 1 but not in year 2. These changing conditions may have had an effect on our results. Third, the species of vegetation differed between sample sites, and we were unable to quantify vegetation density. As other roadside barrier studies have observed or predicted using air quality models, vegetation density has a measurable impact on particle concentrations down-range from roadways [25, 32, 38–40]. Fourth, the distance from the roadway to the sample location was different for the solid barrier site than for the sites with vegetative barriers. Furthermore, the solid barrier site frequently experienced calm wind conditions, and this made it difficult to disentangle the effect of distance to roadway and wind direction category from the effect of the solid barrier. Beyond these noted limitations, this study provides much needed information on the impact of near-road particulate matter concentrations. Although this study was conducted in the southeastern United States, the findings are applicable to other regions with similar temperature as well as a mixture of both deciduous and evergreen vegetation.

## Conclusion

We measured the difference in PNC and BC concentration between roadside sites and comparison sites along major highways in Atlanta, Georgia. We found that wind direction and the presence of either vegetative or solid roadside barriers affected particle concentrations away from the highway, and we observed median particulate concentrations to be lower down-range of vegetative barriers. This effect was more pronounced for BC than for PNC. We used GLMMs to estimate the effect of barriers while controlling for other covariates including wind direction, distance from the sample sites to the roadway, traffic volume on the adjacent highway segment as well as temperature and relative humidity. Modeling results were consistent with vegetative barriers reducing BC concentration away from the highway, and sensitivity analysis suggested this effect was most pronounced when the barrier was downwind of the highway. The effect of vegetative barriers on PNC were not statistically significant but were suggestive of decreasing PNC when the barrier was located downwind from the highway. Modeling results for the effect of the solid barrier were not significant, and due to high correlation between this barrier type and calm wind conditions, were difficult to disentangle from the effect of wind direction. Our results demonstrate several important study design factors to consider in future roadside barrier studies, namely the distance from the roadside to the samplers as well as the distance between roadside and comparison sample sites. These results may be comparable to other urban areas in temperate climates with similar vegetation, traffic volume and vehicle fuel mix. Our findings support the use of roadside barriers to mitigate exposure to particulate TRAP, especially vegetative barriers with higher total surface area than solid barriers to facilitate aerosol dry deposition.

The complete data set for this study is included in the online supporting information (S1 Data) as well as the R code used for dta analysis (S2 Data).

## Supporting information

S1 Data (TXT)

S2 Data (TXT)

## References

[1] HEI. State of Global Air 2019. Boston, MA: Health Effects Institute; 2019.

[2] Kim JB, Prunicki M, Haddad F, Dant C, Sampath V, Patel R, et al. Cumulative lifetime burden of cardiovascular disease from early exposure to air pollution. J Am Heart Assoc. 2020;9(6):e014944-e. doi: 10.1161/JAHA.119.014944 32174249 PMC7335506

[3] Chen Z, Salam MT, Eckel SP, Breton CV, Gilliland FD. Chronic effects of air pollution on respiratory health in Southern California children: findings from the Southern California Children’s Health Study. Journal of Thoracic Disease. 2015;7(1):46–58. doi: 10.3978/j.issn.2072-1439.2014.12.20 25694817 PMC4311073

[4] Costa LG, Cole TB, Dao K, Chang Y-C, Coburn J, Garrick JM. Effects of air pollution on the nervous system and its possible role in neurodevelopmental and neurodegenerative disorders. Pharmacology & Therapeutics. 2020;210:107523. 10.1016/j.pharmthera.2020.107523 PMC7245732 32165138

[5] Peters R, Ee N, Peters J, Booth A, Mudway I, Anstey KJ. Air pollution and dementia: a systematic review. Journal of Alzheimer’s Disease. 2019;70:S145–S63. doi: 10.3233/JAD-180631 30775976 PMC6700631

[6] Liu J, Clark LP, Bechle MJ, Hajat A, Kim S-Y, Robinson AL, et al. Disparities in air pollution exposure in the United States by race/ethnicity and income, 1990–2010. Environmental health perspectives. 2021;129(12):127005. doi: 10.1289/EHP8584 34908495 PMC8672803

[7] Martinez A, de la Rosa R, Mujahid M, Thakur N. Structural racism and its pathways to asthma and atopic dermatitis. Journal of Allergy and Clinical Immunology. 2021;148(5):1112–20. doi: 10.1016/j.jaci.2021.09.020 34743832 PMC9186508

[8] Sohrabi S, Zietsman J, Khreis H. Burden of disease assessment of ambient air pollution and premature mortality in urban areas: the role of socioeconomic status and transportation. International Journal of Environmental Research and Public Health. 2020;17(4):1166. doi: 10.3390/ijerph17041166 32059598 PMC7068272

[9] Khreis H, Nieuwenhuijsen M, Zietsman J, Ramani T. Traffic-Related Air Pollution: Elsevier; 2020 2020. 632 p.

[10] Karner AA, Eisinger DS, Niemeier DA. Near-roadway air quality: synthesizing the findings from real-world data. Environmental science & technology. 2010;44(14):5334–44. doi: 10.1021/es100008x 20560612

[11] Moutinho JL, Liang D, Golan R, Ebelt ST, Weber R, Sarnat JA, et al. Evaluating a multipollutant metric for use in characterizing traffic-related air pollution exposures within near-road environments. Environmental Research. 2020;184:109389. doi: 10.1016/j.envres.2020.109389 32209498 PMC7202092

[12] Patton AP, Perkins J, Zamore W, Levy JI, Brugge D, Durant JL. Spatial and temporal differences in traffic-related air pollution in three urban neighborhoods near an interstate highway. Atmospheric Environment. 2014;99:309–21. doi: 10.1016/j.atmosenv.2014.09.072 25364295 PMC4212216

[13] Sarnat JA, Armistead R, Liang D, Moutinho JL, Golan R, Weber RJ, et al. Developing multipollutant exposure indicators of traffic pollution: the Dorm Room Inhalation to Vehicle Emissions (DRIVE) study. Health Effects Institute; 2018 April 1, 2018. Report No.: 1041–5505 2688–6855. doi: 10.1371/journal.pone.0137789 31872750 PMC7266376

[14] Hilker N, Wang JM, Jeong C-H, Healy RM, Sofowote U, Debosz J, et al. Traffic-related air pollution near roadways: discerning local impacts from background. Atmospheric Measurement Techniques. 2019;12(10):5247–61.

[15] Hu S, Fruin S, Kozawa K, Mara S, Paulson SE, Winer AM. A wide area of air pollutant impact downwind of a freeway during pre-sunrise hours. Atmospheric Environment. 2009;43(16):2541–9. doi: 10.1016/j.atmosenv.2009.02.033 25379010 PMC4219369

[16] Fuller CH, Brugge D. Ambient Combustion Ultrafine Particles and Health: Nova Science Publishers; 2021 2021.

[17] EPA US. Near roadway air pollution and fealth: frequently asked questions. Washington, D.C.: U.S. Environmental Protection Agency, Quality OoTaA; 2014.

[18] World Health Organization. Regional Office for E. Urban green spaces and health. Copenhagen: World Health Organization; 2016 2016. Contract No.: WHO/EURO:2016-3352-43111-60341.

[19] Kumar P, Zavala-Reyes JC, Tomson M, Kalaiarasan G. Understanding the effects of roadside hedges on the horizontal and vertical distributions of air pollutants in street canyons. Environment International. 2022;158:106883. doi: 10.1016/j.envint.2021.106883 34583097

[20] Abhijith KV, Kumar P, Gallagher J, McNabola A, Baldauf R, Pilla F, et al. Air pollution abatement performances of green infrastructure in open road and built-up street canyon environments. Atmospheric Environment. 2017;162:71–86.

[21] Baldauf R. Roadside vegetation design characteristics that can improve local, near-road air quality. Transportation Research Part D: Transport and Environment. 2017;52:354–61. 30057483 10.1016/j.trd.2017.03.013 PMC6060415

[22] Isakov V, Venkatram A, Baldauf R, Deshmukh P, Zhang M. Evaluation and development of tools to quantify the impacts of roadside vegetation barriers on near-road air quality. Int J Environ Pollut. 2017;62(2):127–35. doi: 10.1504/IJEP.2017.10010370 30078956 PMC6071432

[23] Fuller CH, Carter DR, Hayat MJ, Baldauf R, Watts Hull R. Phenology of a vegetation barrier and resulting impacts on near-highway particle number and black carbon concentrations on a school campus. International Journal of Environmental Research and Public Health [Internet]. 2017; 14(2):[13 p.]. doi: 10.3390/ijerph14020160 28208726 PMC5334714

[24] Abhijith KV, Kumar P. Field investigations for evaluating green infrastructure effects on air quality in open-road conditions. Atmospheric Environment. 2019;201:132–47.

[25] Deshmukh P, Isakov V, Venkatram A, Yang B, Zhang KM, Logan R, et al. The effects of roadside vegetation characteristics on local, near-road air quality. Air Quality, Atmosphere & Health. 2019;12:259–70. doi: 10.1016/S1352-2310(02)00923-8 32636958 PMC7339705

[26] Kumar P, Druckman A, Gallagher J, Gatersleben B, Allison S, Eisenman TS, et al. The nexus between air pollution, green infrastructure and human health. Environment International. 2019;133:105181. doi: 10.1016/j.envint.2019.105181 31675531

[27] Dean CB, Nielsen JD. Generalized linear mixed models: a review and some extensions. Lifetime Data Analysis. 2007;13(4):497–512. doi: 10.1007/s10985-007-9065-x 18000755

[28] GDOT. Mileage by route and road system 2021 [March 7, 2022]. Available from: http://www.dot.ga.gov/DriveSmart/Data/Documents/400%20Series/445/445_Report_2020.pdf.

[29] Good N, Mölter A, Peel JL, Volckens J. An accurate filter loading correction is essential for assessing personal exposure to black carbon using an Aethalometer. Journal of Exposure Science & Environmental Epidemiology. 2017;27(4):409–16. doi: 10.1038/jes.2016.71 28000686 PMC5693258

[30] Zhu Y, Hinds WC, Kim S, Shen S, Sioutas C. Study of ultrafine particles near a major highway with heavy-duty diesel traffic. Atmospheric Environment. 2002;36(27):4323–35.

[31] Brantley HL, Hagler GSW, J. Deshmukh P, Baldauf RW. Field assessment of the effects of roadside vegetation on near-road black carbon and particulate matter. Science of The Total Environment. 2014;468–469:120–9. 24008075 10.1016/j.scitotenv.2013.08.001

[32] Hagler GSW, Lin M-Y, Khlystov A, Baldauf RW, Isakov V, Faircloth J, et al. Field investigation of roadside vegetative and structural barrier impact on near-road ultrafine particle concentrations under a variety of wind conditions. Science of The Total Environment. 2012;419:7–15. doi: 10.1016/j.scitotenv.2011.12.002 22281040

[33] Gass K, Balachandran S, Chang HH, Russell AG, Strickland MJ. Ensemble-based source apportionment of fine particulate matter and emergency department visits for pediatric asthma. American Journal of Epidemiology. 2015;181(7):504–12. doi: 10.1093/aje/kwu305 25776011 PMC4447814

[34] Pennington AF, Strickland MJ, Gass K, Klein M, Sarnat SE, Tolbert PE, et al. Source-apportioned PM2.5 and cardiorespiratory emergency department visits: accounting for source contribution uncertainty. Epidemiology. 2019;30(6):789–98. doi: 10.1097/EDE.0000000000001089 31469699 PMC6768727

[35] Watson JG, Chow JC, Lowenthal DH, Antony Chen LW, Shaw S, Edgerton ES, et al. PM2.5 source apportionment with organic markers in the Southeastern Aerosol Research and Characterization (SEARCH) study. Journal of the Air & Waste Management Association. 2015;65(9):1104–18. doi: 10.1080/10962247.2015.1063551 26102211

[36] Fine PM, Sioutas C, Solomon PA. Secondary particulate matter in the United States: insights from the particulate matter supersites program and related studies. Journal of the Air & Waste Management Association. 2008;58(2):234–53. doi: 10.3155/1047-3289.58.2.234 18318339

[37] Mahilang M, Deb MK, Pervez S, Tiwari S, Jain VK. Biogenic secondary organic aerosol formation in an urban area of eastern central India: seasonal variation, size distribution and source characterization. Environmental Research. 2021;195:110802. doi: 10.1016/j.envres.2021.110802 33516684

[38] Mori J, Fini A, Galimberti M, Ginepro M, Burchi G, Massa D, et al. Air pollution deposition on a roadside vegetation barrier in a Mediterranean environment: Combined effect of evergreen shrub species and planting density. Science of The Total Environment. 2018;643:725–37. doi: 10.1016/j.scitotenv.2018.06.217 29957437

[39] Tong Z, Baldauf RW, Isakov V, Deshmukh P, Max Zhang K. Roadside vegetation barrier designs to mitigate near-road air pollution impacts. Science of The Total Environment. 2016;541:920–7. doi: 10.1016/j.scitotenv.2015.09.067 26457737

[40] Zheng T, Jia Y-P, Zhang S, Li X-B, Wu Y, Wu C-L, et al. Impacts of vegetation on particle concentrations in roadside environments. Environmental Pollution. 2021;282:117067. doi: 10.1016/j.envpol.2021.117067 33838442

